# Evaluate Short-Term Outcomes of abciximab in ST-Segment Elevation Myocardial Infarction Patients Undergoing Percutaneous Coronary Intervention: A Meta-Analysis of Randomized Clinical Trials

**DOI:** 10.1155/2022/3911414

**Published:** 2022-05-29

**Authors:** Nan Bai, Ying Niu, Ying Ma, Yao-Sheng Shang, Peng-Yu Zhong, Zhi-Lu Wang

**Affiliations:** ^1^The First Clinical Medical College of Lanzhou University, Lanzhou, China; ^2^Department of Cardiology, The First Hospital of Lanzhou University, Lanzhou, China

## Abstract

**Objectives:**

This meta-analysis was to verify the short-time efficacy and safety of abciximab in patients with ST-segment elevation myocardial infarction (STEMI) undergoing percutaneous coronary intervention (PCI).

**Background:**

Abciximab has long-term efficacy in patients with STEMI undergoing PCI, but the short-term efficacy is still controversial.

**Methods:**

We conducted a systematic review and meta-analysis compared with or without abciximab in patients with STEMI undergoing PCI. The relevant randomized controlled trials were included by searching PubMed, EMBASE, Cochrane Library, and Web of Science databases and other sources. The relative risk (RR) and 95% confidence intervals (CI) of outcomes were calculated by the fixed-effects model.

**Results:**

Ten randomized controlled trials with 5008 patients met inclusion criteria. There were no significant differences in risk of all-cause death at 30-day (RR 0.79, CI 0.55–1.12, *P*=0.18), major bleeding (1.37, 0.93–2.03, *P*=0.11), and transfusion (1.23, 0.94–1.61, *P*=0.13) between the two groups. However, there were significant differences in risk of all-cause death at 6 months (0.57, 0.36–0.90, *P*=0.02), recurrent myocardial infarction (0.55, 0.33–0.92, *P*=0.02), repeat revascularization (0.58, 0.43–0.78, *P*=0.0004), final TIMI flow <3 (0.77, 0.62–0.96, *P*=0.02), minor bleeding (1.29, 1.02–1.63, *P*=0.04), and thrombocytopenia (2.04, 1.40–2.97, *P*=0.0002).

**Conclusions:**

The application of abciximab can lead to a lower risk of reinfarction, revascularization, and all-cause death at 6 months, but a higher risk of minor bleeding, and thrombocytopenia.

## 1. Introduction

As the first glycoprotein (GP) IIb/IIIa inhibitor (GPI) studied, abciximab inhibits thrombus formation by blocking the binding of fibrinogen, von Willebrand factor, or other ligands to IIb/IIIa receptors [1]. Abciximab has a strong antiplatelet aggregation effect and can exert the maximal antiplatelet effect 10 minutes after its bolus administration [2]. In the 2000s, it was used as an antiplatelet drug for patients with non-ST-segment elevation myocardial infarction and ST-segment elevation myocardial infarction (STEMI) undergoing percutaneous coronary intervention (PCI) [3, 4]. In addition, abciximab can bind to the vitronectin receptor and may have non-GP IIb/IIIa-dependent anti-inflammatory properties, and its importance in clinical outcomes is not completely understood [1, 5].

Animal experiments and early clinical trials have demonstrated that abciximab can provide potent action of antiaggregation [6, 7]. Subsequently, various studies on the efficacy of abciximab in patients undergoing PCI were carried out. Several trials have consistently concluded that abciximab can bring long-term clinical benefits in reducing composite ischemic endpoints, including mortality. The long-term refers to a follow-up period of at least 1 year and sustained out to 3 years [8–11]. However, the short-term efficacy of abciximab in patients with STEMI undergoing PCI is controversial in the follow-up period of 30-day or 6-month. Two meta-analyses showed that abciximab significantly reduced the incidence of the primary endpoint at 30 days and 6 months [12, 13]. Another study showed the primary endpoint of all-cause death, myocardial infarction (MI), or urgent revascularization decreased significantly only at 30 days, but not at 6 months [14]. Even trials have shown there is no clinical benefit with the application of abciximab regardless of at 30 days or 6 months [15, 16]. Some trials have shown that the short-term benefit of abciximab is affected by thienopyridines or fibrinolysis [17, 18]. Obviously, this issue needs clarification further.

Therefore, this meta-analysis was to verify the short-time efficacy and safety of abciximab in patients with STEMI undergoing PCI. The results showed that despite the increased risk of bleeding and thrombocytopenia, abciximab can provide short-term benefits for patients with STEMI, and the adverse reactions of the drug can be weakened by selecting thienopyridines.

## 2. Methods

The literature was searched by PubMed, Embase, Cochrane Library, Web of Science databases, and clinicalTrials.gov from inception to 17 April 2022. The study only included randomized controlled trials comparing short-term efficacy and safety of abciximab in patients with STEMI undergoing PCI. The keywords were as follows: “abciximab,” “ST-segment elevation myocardial infarction,” and “randomized controlled trial” (Supplementary Table 1). There were no language and year of publication restrictions. An update reminder for PubMed was created to keep up with the latest research. The inclusion criterion of the study met the following requirements: (1) STEMI defined clinically with persistent myocardial ischemia symptoms and electrocardiographic evidence but without angiographic selection criteria, (2) reperfusion therapy with PCI, (3) comparison of patients with or without abciximab, and (4) the trials that reported the risk of mortality at 30 days or 6 months. The exclusion criterion of the study included nonrandomized controlled trial and observation studies, as well as patients with non-STEMI. The title, abstract, and full text were independently read to determine whether the trials met the inclusion and exclusion criteria by 2 investigators (Bai N and Niu Y). The discrepancy was solved by consultation with the third party (Ma Y, Shang YS, and Zhong PY). The quality of each randomized controlled trial was evaluated according to the Cochrane tool of Collaboration for assessing the risk of bias, and the Grades of Recommendations Assessment, Development, and Evaluation (GRADE) was conducted to evaluate the quality of each outcome [19, 20]. The meta-analysis protocol was registered in PROSPERO (CRD42020211386).

Data extraction and analysis followed the Preferred Reporting Items for Systematic Reviews and Meta-analysis (PRISMA) statement [21], and intention-to-treat analysis was employed. The baseline characteristics of patients and trials were extracted by 2 researchers independently, and the divergences were resolved through negotiation (Wang ZL). The primary efficacy outcomes consisted of all-cause death at 30 days and 6 months, recurrent MI, repeat revascularization, and final thrombolysis in myocardial infarction (TIMI) flow <3. The primary safety outcomes included major bleeding, minor bleeding, thrombocytopenia, and transfusion. The major bleeding, minor bleeding, thrombocytopenia, and transfusion were defined based on the definition used in the clinical studies included.

This meta-analysis was used by Review Manager Version 5.4 software (The Nordic Cochrane Center, Copenhagen, Denmark) and Stata 14.1 (Statacorp LP, College Station, Texas, USA), and the Cochrane *Q* statistic with Pearson chi-square test and the Higgins *I*^2^ test were performed to assess heterogeneity in Review manager. If there was substantial heterogeneity (*I*^2^ ≥ 50% and *P*_heterogeneity_ < 0.1), the sensitivity analysis would be performed. Revman software was used to identify any single trial that may affect the overall results and cause substantial heterogeneity. If heterogeneity was reduced after excluding one trial (*I*^2^ < 50% and *P*_heterogeneity_ > 0.1), the trial would be regarded as the source of heterogeneity. Therefore, the sensitivity analysis would be completed by the “leave-one-out.” If the sensitivity analysis cannot reduce heterogeneity by using the fixed-effect model, the risk ratio (RR) and 95% confidence interval (CI) of each result are calculated using the random effect model. The meta-regression was performed with Stata 14.1 (Statacorp LP, College Station, Texas, USA) to explore potential effect modifiers. Subgroup analyses were performed according to the antiplatelet strategy. Two-tailed *P* values were exploited for all results, and statistical significance was set at *P* < 0.05. Trial Sequential Analysis (TSA) version 0.9.5.10 software was used to estimate the sample size of statistical differences in each outcome (based on an *α* of 0.05 and a power of 0.8). Moreover, Egger's and Begg's tests, as well as visual inspection of funnel plots, were employed to assess publication bias.

## 3. Results

### 3.1. Search Results and Study Characteristics

**Figure 1:**
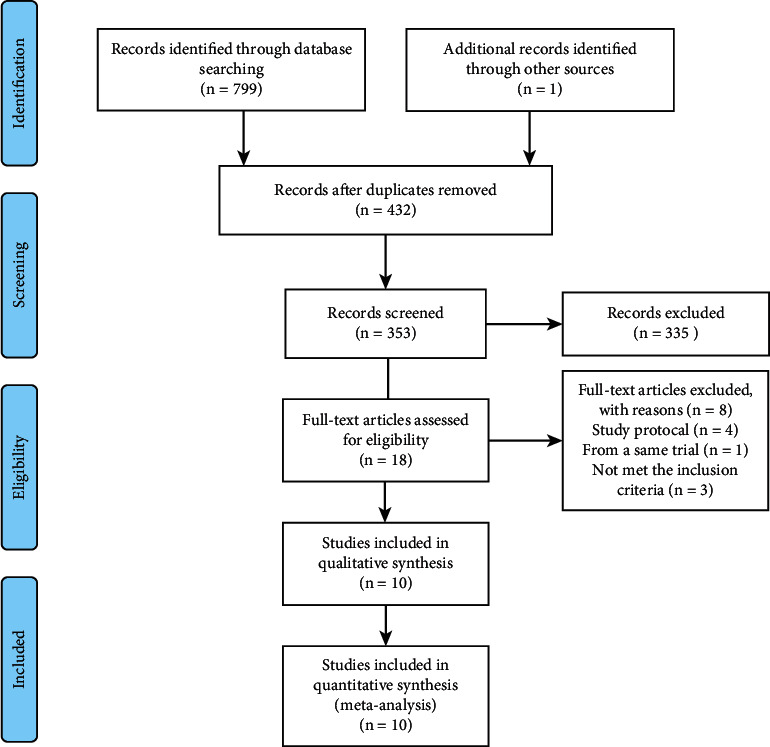
Flow diagram of literature search.

A total of 799 articles are included which are extracted from the above medical databases. Another article named “Deferred stenting in patients with anterior wall STEMI” came from the clinicalTrials.gov, and it is still recruiting patients (NCT03744000). Finally, 18 articles are initially identified by reading title and abstract (Figure 1). After reading the full text, ten randomized controlled trials with a total of 5008 patients with STEMI undergoing PCI were determined [3, 4, 22–29]. Among them, 2518 patients were divided into abciximab group and 2490 patients were divided into control group. The characteristics and outcomes of trials included are shown (Tables 1, 2). All the patients included were adults (>18 years old). The average age of the patients included in the study was 61.06 years old. Meanwhile, 75.04% patients were males, patients with hypertension accounted for 52.77%, and patients with diabetes mellitus accounted for 17.15%. The proportion of patients with dyslipidemia was 42.71%, who included patients with hyperlipidemia and hypercholesterolemia. The onset time of acute myocardial infarction in the included patients ranged from 6 hours to 48 hours. In 8 trials, patients randomized to the abciximab group received the drug as a bolus of 0.25 mg/kg body weight, followed by a 12 h infusion at a rate of 0.125 *μ*g/kg/min [4, 22–24, 26–29]. In the other 2 trials, the maintenance dose of abciximab was 10 *μ*g/min while the initial dose was the same [3, 25]. Unfractionated heparin and aspirin were used in all studies, but the specific usage and dosage were not exactly the same in different trials. Clopidogrel and ticlopidine were used selectively. The duration of follow-up in the trials included was from in-hospital to one year. The baseline demographic and clinical characteristics of patients are reported (Table 3). Previous revascularization refers to previous treatment with percutaneous transluminal coronary angioplasty or coronary artery bypass grafting. Multivessel lesion is defined as the number of vessel with obvious stenosis or occlusion >1.

### 3.2. Quality Assessment

All studies in this meta-analysis were randomized controlled trials, and the risk of bias for each trial was assessed by the Cochrane tool of Collaboration. The results of the quality assessment are presented (Supplementary Figure 1). The risk of bias in selection, detection, and reporting was low in all trials, but the risk of bias for performance was high in 7 of 10 trials because 5 of 7 trials were nonblind and 2 were single-blind. In addition, a high risk of bias for attrition was found in 5 trials because of incomplete data on clinical outcomes.

The assessment of the evidence quality for each outcome is shown (Supplementary Table 2). The evidence quality of outcomes was determined to be moderate for major bleeding, minor bleeding, thrombocytopenia, and high for all-cause death at 30 days and 6 months, recurrent MI, repeat revascularization, final TIMI flow <3, and transfusion.

The TSA of each outcome is conducted (Supplementary Figure 2). The curve of the all-cause death at 6 months reached both the conventional boundary and TSA boundary, while the curve of repeat revascularization and thrombocytopenia exceeded the expected sample size. The curve of the recurrent MI, final TIMI flow <3, and minor bleeding met the conventional boundary only, and the curve of the all-cause death at 30 days, major bleeding, and transfusion did not meet the conventional boundary, TSA boundary, and anticipated sample size. There was no publication bias, and the results showed that the distribution is symmetrical in the funnel plot, and the *P* value of Begg's and Egger's is >0.05 in all outcomes (Supplementary Figures 3 and 4).

### 3.3. The Efficacy Outcomes

**Figure 2:**
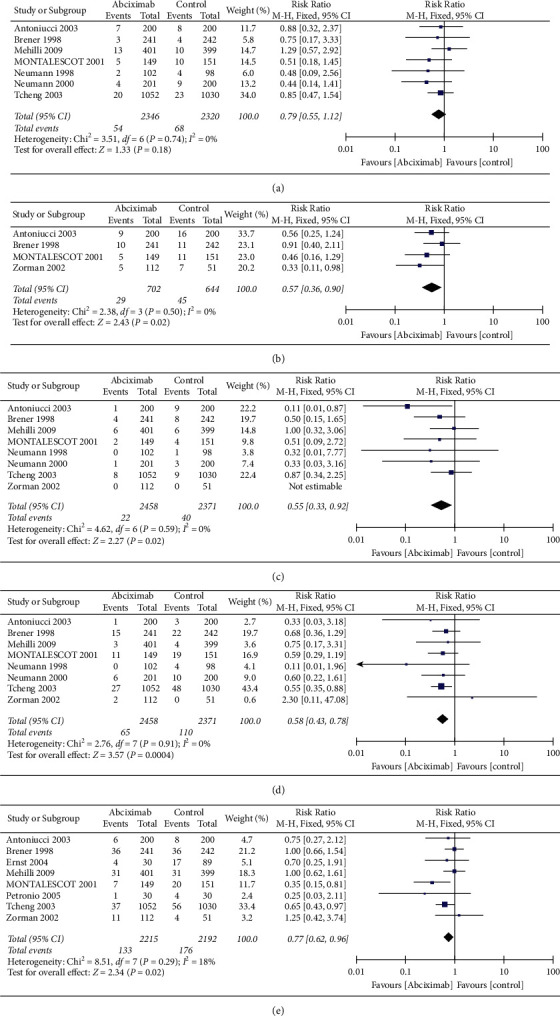
Comparison of primary efficacy outcomes between the abciximab and control groups. (a) All-cause death at 30-day, (b) all-cause death at 6 month, (c) recurrent MI, (d) repeat revascularization, and (e) final TIMI flow <3.

The risk of all-cause death at 30 days is presented in 7 trials (Figure 2(a)), and it is lower in the abciximab group, but there was no significant difference and heterogeneity between the two groups (2.3% vs. 2.9%, RR 0.79, 0.55–1.12, *P*=0.18, *I*^2^ = 0%, *P*_Heterogeneity_=0.74). Four of the 10 trials reported the risk of all-cause death at 6 months, and the events in the abciximab group significantly reduced (4.1% vs. 7.0%, RR 0.57, 0.36–0.90, *P*=0.02, *I*^2^ = 0%, *P*_Heterogeneity_=0.50) (Figure 2(b)).

The incidence of recurrent MI and repeat revascularization events are reported in 8 trials (Figure 2(c), 2(d)). The incidence of recurrent MI (0.9% vs. 1.7%, RR 0.55, 0.33–0.92, *P*=0.02, *I*^2^ = 0%, *P*_Heterogeneity_=0.59) and repeat revascularization events (2.6% vs. 4.6%, RR 0.58, 0.43–0.78, *P*=0.0004, *I*^2^ = 0%, *P*_Heterogeneity_=0.91) were significantly lower in the abciximab group than those in the control group. In addition, 8 trials mentioned the outcome of final TIMI flow <3, and there is a significant difference between the two groups (6.0% vs. 8.0%, RR 0.77, 0.62–0.96, *P*=0.02*I*^2^ = 18%, *P*_Heterogeneity_=0.29) (Figure 2(e)).

### 3.4. The Safety Outcomes and Sensitivity Analysis

**Figure 3:**
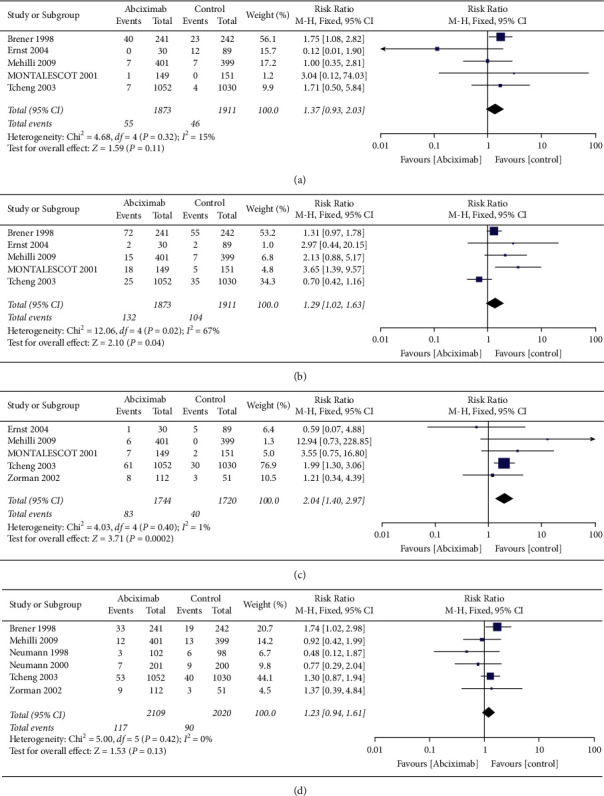
Comparison of primary safety outcomes between the abciximab and control groups. (a) Major bleeding, (b) minor bleeding, (c) thrombocytopenia, and (d) transfusion.

Five trials reported the risk of major bleeding and minor bleeding. The result reveals that the risk of major bleeding events was similar between the two groups, with mild heterogeneity but without significant difference (2.9% vs. 2.4%, RR 1.37, 0.93–2.03, *P*=0.11, *I*^2^ = 15%, *P*_Heterogeneity_=0.32) (Figure 3(a)). The risk of minor bleeding events increased in the abciximab group compared with the control group (7.0% vs. 5.4%, RR 1.29, 1.02–1.63, *P*=0.04, *I*^2^ = 67%, *P*_Heterogeneity_=0.02) (Figure 3(b)). However, there was moderate heterogeneity in the outcome of minor bleeding. One trial produced heterogeneity was identified by sensitivity analysis, the heterogeneity of minor bleeding outcomes was reduced after excluding the results of this trial (*I*^*2*^ = 42%, *P*_Heterogeneity_=0.16) [26], and there is still a significant difference between the two groups (13.0% vs. 7.8%, RR 1.59, 1.22–2.09, *P*=0.0007) (Supplementary Figure 5). Five trials declared the thrombocytopenia events, and the incidence of thrombocytopenia outcome is significantly higher in the abciximab group (4.8% vs. 2.3%, RR 2.04, 1.40–2.97, *P*=0.0002, *I*^2^ = 1%, *P*_Heterogeneity_=0.40) (Figure 3(c)). In addition, there is no significant difference in the incidence of transfusion between the two groups in 6 trials (5.5% vs. 4.5%, RR 1.23, 0.94–1.61, *P*=0.13, *I*^2^ = 0%, *P*_Heterogeneity_=0.42) (Figure 3(d)).

### 3.5. The Meta-Regression Analysis and Subgroup Analyses

The meta-regression analysis is performed according to the year of publication, sample size (the total number of patients over 1000 is defined as a large sample trial, while that less than 400 is defined as a small sample trial, and that between 400 and 1000 is defined as a medium sample trial), patient classification (patients with cardiac shock were divided into the high-risk group and patients without cardiac shock were divided into the low-risk group), and timing of application of abciximab (before coronary angiography or before PCI but after coronary angiography). Sample size may be a factor that results in the heterogeneity of minor bleeding (Supplementary Figures 6A–6D).

**Figure 4:**
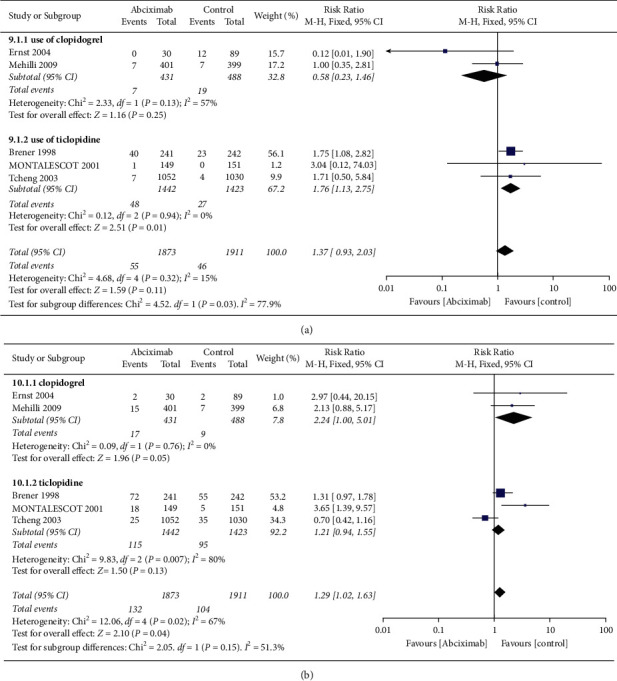
Subgroup analyses in (a) major bleeding and (b) minor bleeding between clopidogrel and ticlopidine groups.

The subgroup analyses are performed in major bleeding and minor bleeding according to the antiplatelet strategy (combined with clopidogrel or ticlopidine) (Figures 4(a) and 4(b)). In major bleeding, there was no significant difference in risk of major bleeding between the two groups in patients with clopidogrel (1.6% vs. 3.9%, RR 0.58, 0.23–1.46, *P*=0.25, *I*^2^ = 57%, *P*_Heterogeneity_ = 0.13). Instead, there was a significant difference in patients with ticlopidine (3.3% vs. 1.9%, RR 1.76, 1.13–2.75, *P*=0.01, *I*^2^ = 0%, *P*_Heterogeneity_=0.94). Meanwhile, there were differences between the two strategies with statistically significant (*I*^2^ = 77.9%, *P*_interaction_=0.03) (Figure 4(a)). In minor bleeding, no significant difference was found in the clopidogrel group (3.9% vs. 1.8%, RR 2.24, 1.00–5.01, *P*=0.05, *I*^2^ = 0%, *P*_Heterogeneity_=0.76) and the ticlopidine group (8.0% vs. 6.7%, RR 1.21, 0.94–1.55, *P*=0.13, *I*^2^ = 80%, *P*_Heterogeneity_=0.007). In addition, the difference between the two groups was not statistically significant (*I*^2^ = 51.3%, *P*_interaction_=0.15) (Figure 4(b)).

When only double-blind trials were included [4, 22, 24], the application of abciximab still increased the risk of minor bleeding, but with moderate heterogeneity (13.3% vs. 8.5%, RR 1.57, 1.20–2.07, *P*=0.001, *I*^2^ = 58%, *P*_heterogeneity_=0.09) (Supplementary Figures 7A–7F).

## 4. Discussion

The meta-analysis demonstrates the relative risk reduction of all-cause death at 30 days and 6 months was 21% and 43%, respectively. Although the antiaggregation effect of abciximab on platelets can last for 15 days after administration [30], the period of clinical benefit can be as long as months or even years [9–11]. Clinical studies have shown that distal embolization is associated with larger infarct size, lower left ventricular ejection fraction, and higher risk of mortality [31]. The application of abciximab can reduce the formation of distal embolization and improve myocardial perfusion, thereby bringing about long-term benefits [18, 32]. However, less than 20% of patients with distal embolization can be recognized during the angiography [31]. Combined with the more common problems of distal embolization in patients with anterior or multivessel diseases and previous MI, the application of abciximab in this population may be beneficial [31]. In addition, it is unclear how it is affected by the dual antiplatelet drugs currently used. Therefore, it is necessary to further study whether the combination of abciximab on the basis of dual antiplatelet will benefit from patients who may have distal embolization. The risk ratio of recurrent MI, repeat revascularization, and final TIMI flow <3 were significantly reduced by 45%, 42%, and 23%, respectively, which had significant clinical benefits. However, the curve of recurrent MI and final TIMI flow (<3) did not exceed the TSA boundary, which demonstrates more randomized controlled trials are needed to meet the anticipated sample size.

The study demonstrates that, compared with the control group, the risk ratio of major bleeding in the abciximab group was increased by 37%. Similar to the result of another meta-analysis, abciximab can increase the likelihood of major bleeding [12]. However, the results were not statistically significant, which may be related to the different definitions of major bleeding in each trial. Ticlopidine resulted in a higher incidence of major bleeding compared with clopidogrel based on subgroup analysis, which was consistent with the results of other studies [33, 34]. In fact, ticlopidine was limited by its serious side effects, such as neutropenia, thrombotic thrombocytopenic purpura, and bone marrow aplasia [35]. Considering moderate heterogeneity was observed in minor bleeding (*I*^2^ = 67%), the sensitivity analysis was further performed and the CADILLAC trial is considered to be the main cause of heterogeneity [26]. After removal, the heterogeneity of the result was reduced to a mild level (42%). Combined with *P*_heterogeneity_ > 0.1, the results indicated that the use of abciximab was associated with a higher risk of minor bleeding (*P*=0.0007). The possible reason for the heterogeneity caused by the CADILLAC trial was that in the other 4 trials [4, 22–24], the incidence of events in the abciximab group was not less than that in the control group, while in the CADILLAC trial [26], the event incidence of the control group was higher. To find out other possible causes resulting in heterogeneity, meta-regression and subgroup analyses were carried out. The results showed that, except for sample size, no other possible sources of heterogeneity were observed, such as whether the patient with or without a high risk of ischemia, providing abciximab earlier or later, and the use of clopidogrel or ticlopidine to antiplatelet. Due to the lack of subgroup data on factors such as age, gender, infarct vessel, Killip class, and left ventricular ejection fraction, it is impossible to verify whether the relevant factors are responsible for heterogeneity. In addition, there were only five trials with 3784 patients that included minor bleeding [4, 22–24, 26], three of which were double-blind designs [4, 22, 24]. The limited number of trials and patients may lead to heterogeneity. The incidence of transfusion was higher in the abciximab group, but there was no significant difference. Although the indications of transfusion were not stated in most of the trials included, the transfusion was directly associated with major bleeding in the trial conducted by Ernst et al. [23]. In this meta-analysis, the incidence of thrombocytopenia caused by abciximab doubled. This study reported an absolute risk increase of 2.5% in thrombocytopenia, which translates into a number needed to harm of 40. In other words, for every 40 patients treated, there will be 1 case of thrombocytopenia caused by the use of abciximab. However, discontinuation should be considered only when severe thrombocytopenia occurs with a platelet count below 20,000/*μ*L [36].

Since the results of this meta-analysis come from specific situations and populations, the conclusions of this study need to be applied carefully. On the one hand, the study population were all patients with STEMI, and the efficacy of the drug may be different from patients with unstable angina or non-ST-segment elevation myocardial infarction. The results are different from studies of patients with these diseases [15, 37], and the application of abciximab was recommended only in this cohort for early coronary intervention [38]. On the other hand, the molecular structure of abciximab is different from the other two GPIs (tirofiban and eptifibatide) [39]. Some trials in which tirofiban was not inferior to abciximab have drawn different conclusions [40, 41]. Therefore, these conclusions are not easy to be extended to the above population or other GPIs.

### 4.1. Limitations

The present meta-analysis of randomized clinical trials may have some limitations. First of all, there are inevitable differences between trials, such as lengths of the onset of symptoms, the design of the primary outcomes, and the definition of outcomes. Secondly, double-blind design was only implemented in 3 trials, and the other trials were designed as single-blind or open-label, which affected the quality of the study because of the increased risk of bias. In addition, in half of the trials, part of the data on the clinical outcomes mentioned in the text was incomplete, and the clinical outcomes involved in this study were not available from all trials. Thirdly, according to the results of TSA, the outcomes of all-cause death at 30 days, final TIMI flow <3, major bleeding, minor bleeding, and transfusion did not surpass the TSA boundary, which may lead to false-positive results. Finally, the trials included in this meta-analysis are relatively old, and there is insufficient evidence of the effect of abciximab in a dual antiplatelet context. Therefore, more clinical trials are needed to confirm the efficacy of the drug in this era.

## 5. Conclusions

This systematic review and meta-analysis demonstrates that abciximab was associated with a lower risk of short-term all-cause death, recurrent MI, repeat revascularization, and better myocardial perfusion in patients with STEMI undergoing PCI but a higher risk of minor bleeding and thrombocytopenia. The risk of major bleeding may be relieved by choosing clopidogrel rather than ticlopidine, and the continued use of abciximab will not be affected if there is no severe thrombocytopenia.

## Figures and Tables

**Table 1:** Characteristics of the trials included.

Study	Journal	Publication year	No. of centers	No.of patients	Patients	Randomization	Types of stent	Time of abciximab	Follow-up duration
Antoniucci et al. [27]	JACC	2003	—	400	With CS	Abciximab (*n* = 200) vs. placebo (*n* = 200)	BMS	Before PCI	30 days 6 months
Brener et al. [22]	Circulation	1998	36	483	No CS	Abciximab (*n* = 241) vs. placebo (*n* = 242)	BMS	Before angiography	30 days 6 months
Ernst et al. [23]	JACC	2004	Single-center	119	No CS	Abciximab (*n* = 30) vs. placebo (*n* = 89)	—	Before PCI	In-hospital 30 days
Mehilli et al. [24]	Circulation	2009	5	800	No CS	Abciximab (*n* = 401) vs. placebo (*n* = 399)	BMS DES	Before angiography	30 days
Montalescot et al. [4]	N Engl J Med	2001	26	300	With CS	Abciximab (*n* = 149) vs. placebo (*n* = 151)	BMS	Before angiography	30 days 6 months
Neumann et al. [25]	Circulation	1998	—	200	—	Abciximab (*n* = 102) vs. placebo (*n* = 98)	BMS	—	14 days 30 days
Neumann et al. [3]	JACC	2000	—	401	—	Abciximab (*n* = 201) vs. placebo (*n* = 200)	BMS	—	30 days 1 year
Petronio et al. [28]	Am Heart J	2005	—	60	No CS	Abciximab (*n* = 30) vs. placebo (*n* = 30)	BMS	Before PCI	30 days 6 months
Tcheng et al. [26]	Circulation	2003	76	2082	No CS	Abciximab (*n* = 1052) vs. placebo (*n* = 1030)	BMS	—	30 days 1 year
Zorman et al. [29]	Am J Cardiol	2002	—	163	With CS	Abciximab (*n* = 112) vs. placebo (*n* = 51)	BMS	Both	In-hospital 6 months

CS: cardiac shock; BMS: bare-metal stent; DES: drug-eluting stent; PCI, percutaneous coronary intervention.

**Table 2:** Outcomes of the trials included.

Study	The primary outcomes	The secondary outcomes	The safety outcomes
Antoniucci et al. [27]	Composite of death from any cause, reinfarction, TVR, and stroke within one month	ST-segment reduction; postprocedural cTFC; infarct size at one month; all-cause death, reinfarction, a composite of death and reinfarction, TVR at 6 months	—
Brener et al. [22]	Death, reinfarction, or any TVR at 6 months	Early death, reinfarction, or urgent TVR	Bleeding
Ernst et al. [23]	Recurrent MI	—	Bleeding
Mehilli et al. [24]	Infarct size measured by SPECT	All-cause death, recurrent MI, stroke, urgent TVR, the in-hospital incidence of bleeding complications	Bleeding
Montalescot et al. [4]	composite of death, reinfarction, or urgent TVR	composite of death, reinfarction, or any TVR at 30 days and 6 months	Bleeding
Neumann et al. [25]	The differences in papaverine-induced coronary flow velocity and in wall motion index between the initial and 14-day follow-up	The adverse cardiac events (death, nonlethal reinfarction, and TVR) at 30 days	—
Neumann et al. [3]	Late loss; composite of death, recurrent MI, and TVR	Nonfatal death, recurrent MI, and TVR	—
Petronio et al. [28]	Prevalence of 6-month left ventricular remodeling	Prevalence of angiographic no-reflow during angioplasty; the final cTFC; the percent change in LVEDV at 6 months	—
Tcheng et al. [26]	Composite of death, reinfarction, urgent repeat TVR, or disabling stroke at 30 days/12 months	—	Bleeding
Zorman et al. [29]	Composite of death, heart failure, and/or urgent TVR	—	—

TVR: target vessel revascularization; cTFC: corrected thrombolysis in myocardial infarction (TIMI) frame count; MI: myocardial infarction; SPECT: single-photon emission computed tomography; LVEDV: left ventricular end-diastolic volume.

**Table 3:** Baseline demographic and clinical characteristics of patients included.

	Antoniucci 2003		Brener 1998		Ernst 2004		Mehilli 2009		Montalescot 2001		Neumann 1998		Neumann 2000		Petronio 2005		Tcheng 2003		Zorman 2002	
	A	C	A	C	A	C	A	C	A	C	A	C	A	C	A	C	A	C	A	C
Patients (n)	200	200	241	242	30	89	401	399	149	151	102	98	201	200	30	30	1052	1030	112	51
Age (mean) (years)	64	63	60	62	62.5	60.6	62.4	61.8	59.6	62.1	60.6	60.2	61.3	60.1	60	58.5	60.0	59.0	60.5	63
Male (%)	76	79	73	72	67.0	78.3	76.0	73.0	85.2	78.2	76.0	78.0	74.1	77.0	80	85.0	74.0	71.9	76.0	61
Hypertension (%)	46	47	46	50	43.0	35.0	70.0	71.0	34.2	41.1	62.0	63.0	65.7	62.0	—	—	48.7	47.5	56.5	61
Diabetes (%)	17	19	23	22	20.0	9.0	19.0	16.0	15.4	19.9	14.0	13.0	17.4	12.5	17	20.0	17.5	15.7	20.5	15
Previous MI (%)	10	12	17	21	7.0	6.3	10.0	11.0	14.1	7.3	—	—	—	—	—	—	14.5	12.9	12.5	14
Previous TVR (%)	6	9	14	14	10.0	9.0	4.0	2.0	28.1	23.3	8.0	13.0	11.5	10.5	—	—	14.2	12.0	4.5	8.0
Smoking (%)	39	41	41	41.0	52.0	43.3	42.0	41.0	45.0	39.7	61.0	47.0	42.8	43.5	57	52.0	42.8	43.5	21.0	31
Dyslipidemia (%)	40	39	—	—	28.0	28.0	42.0	44.0	39.6	37.1	65.0	62.0	45.8	40.5	—	—	39.2	36.6	53.5	43
Multivessel disease (%)	54	57	57	62.0	—	—	—	—	—	—	66.0	57.0	62.2	58.0	47	45.0	49.1	48.4	55.5	65
Aspirin	+	+	+	+	+	+	+	+	+	+	+	+	+	+	+	+	+	+	+	+
Unfractionated heparin	+	+	+	+	+	+	+	+	+	+	+	+	+	+	+	+	+	+	+	+
Clopidogrel	+	+	0	0	+	+	+	+	0	0	0	0	0	0	+	+	0	0	0	0
Ticlopidine	+	+	+	+	0	0	0	0	+	+	+	+	+	+	0	0	+	+	0	0

A, the abciximab group; C, the control group; MI, myocardial infarction.

## Data Availability

Data sharing is not applicable to this article as no datasets were generated or analyzed during the current study.

## Supplementary Materials

Supplementary Materials Supplementary Figure 1: assessment of the risk of bias in each randomized controlled trial included. Supplementary Figure 2: The size of the information required for each outcome. (A) All-cause death at 30 days, (B) all-cause death at 6 months, (C) recurrent MI, (D) repeat revascularization, (E) final TIMI flow <3, (F) major bleeding, (G) minor bleeding, (H) thrombocytopenia, and (I) transfusion. Supplementary Figure 3: funnel plot of each outcome. (A) All-cause death at 30 days, (B) all-cause death at 6 months, (C) recurrent MI, (D) repeat revascularization, (E) final TIMI flow <3, (F) major bleeding, (G) minor bleeding, (H) thrombocytopenia, and (I) transfusion. Supplementary Figure 4: Begg's and Egger's tests in Stata of each outcome. (A) All-cause death at 30 days, (B) all-cause death at 6 months, (C) recurrent MI, (D) repeat revascularization, (E) final TIMI flow <3, (F) major bleeding, (G) minor bleeding, (H) thrombocytopenia, and (I) transfusion. Supplementary Figure 5: sensitivity analysis of minor bleeding. Supplementary Figure 6: the meta-regression analysis of minor bleeding according to (A) publication year, (B) sample size, (C) patient classification, and (D) timing of application of abciximab. Supplementary Figure 7: comparison of the endpoints between the abciximab and control groups of the double-blind studies. (A) All-cause death at 30 days, (B) recurrent myocardial infarction, (C) repeat revascularization, (D) final TIMI flow <3, (E) major bleeding, and (F) minor bleeding. Supplementary Table 1: search strategy. Supplementary Table 2: Summary of GRADE evidence quality for outcomes.
