# Supplementary material for: Evaluate Short-Term Outcomes of abciximab in ST-Segment Elevation Myocardial Infarction Patients Undergoing Percutaneous Coronary Intervention: A Meta-Analysis of Randomized Clinical Trials
Source: J Interv Cardiol. 2022 May 29;2022:3911414. doi: 10.1155/2022/3911414 (PMC9168187; doi:10.1155/2022/3911414)
Supplement: Supplementary Materials — Supplementary Figure 1: assessment of the risk of bias in each randomized controlled trial included. Supplementary Figure 2: The size of the information required for each outcome. (A) All-cause death at 30 days, (B) all-cause death at 6 months, (C) recurrent MI, (D) repeat revascularization, (E) final TIMI flow <3, (F) major bleeding, (G) minor bleeding, (H) thrombocytopenia, and (I) transfusion. Supplementary Figure 3: funnel plot of each outcome. (A) All-cause death at 30 days, (B) all-cause death at 6 months, (C) recurrent MI, (D) repeat revascularization, (E) final TIMI flow <3, (F) major bleeding, (G) minor bleeding, (H) thrombocytopenia, and (I) transfusion. Supplementary Figure 4: Begg's and Egger's tests in Stata of each outcome. (A) All-cause death at 30 days, (B) all-cause death at 6 months, (C) recurrent MI, (D) repeat revascularization, (E) final TIMI flow <3, (F) major bleeding, (G) minor bleeding, (H) thrombocytopenia, and (I) transfusion. Supplementary Figure 5: sensitivity analysis of minor bleeding. Supplementary Figure 6: the meta-regression analysis of minor bleeding according to (A) publication year, (B) sample size, (C) patient classification, and (D) timing of application of abciximab. Supplementary Figure 7: comparison of the endpoints between the abciximab and control groups of the double-blind studies. (A) All-cause death at 30 days, (B) recurrent myocardial infarction, (C) repeat revascularization, (D) final TIMI flow <3, (E) major bleeding, and (F) minor bleeding. Supplementary Table 1: search strategy. Supplementary Table 2: Summary of GRADE evidence quality for outcomes. [file 3911414.f1.zip › 3911414.f1/Supplemental 1 (clean version).doc]

Supplemental

Supplementary Figure 1. Assessment for the risk of bias in each randomized controlled trial included.

Supplementary Figure 2. The size of information required for each outcome. (A) all-cause death at 30-day, (B) all-cause death at 6-month, (C) recurrent MI, (D) repeat revascularization, (E) final TIMI flow < 3, (F) major bleeding, (G) minor bleeding, (H) thrombocytopenia, (I) transfusion.

Supplementary Figure 3. Funnel plot of each outcome. (A) all-cause death at 30-day, (B) all-cause death at 6-month, (C) recurrent MI, (D) repeat revascularization, (E) final TIMI flow <3, (F) major bleeding, (G) minor bleeding, (H) thrombocytopenia, (I) transfusion.

Supplementary Figure 4. The Begg’s and Egger’s Test in STATA of each outcomes. (A) all-cause death at 30-day, (B) all-cause death at 6-month, (C) recurrent MI, (D) repeat revascularization, (E) final TIMI flow < 3, (F) major bleeding, (G) minor bleeding, (H) thrombocytopenia, (I) transfusion.

Supplementary Figure 5. Sensitivity analysis of minor bleeding.

Supplementary Figure 6. The meta-regression analysis of minor bleeding according to (A) publication year, (B) sample size, (C) patient classification and (D) timing of application of abciximab.

Supplementary Figure 7. Comparison of the endpoints between the abciximab and control groups of the double blind studies. (A) all-cause death at 30-day, (B) recurrent myocardial infarction, (C) repeat revascularization, (D) final TIMI flow < 3, (E) major bleeding, (F) minor bleeding.

Supplementary Table 1. Search strategy.

1. Pubmed search strategy

| # | Searches | Results |
| --- | --- | --- |
| #1 | Search: ((((((abciximab) OR (c7E3 Fab)) OR (Chimeric 7E3 Fab)) OR (Fab, Chimeric 7E3)) OR (ReoPro)) OR (Clotinab)) OR (CentoRx) | 2831 |
| #2 | Search: (((ST Elevation Myocardial Infarction) OR (ST Segment Elevation Myocardial Infarction)) OR (ST Elevated Myocardial Infarction)) OR (STEMI) | 30324 |
| #3 | Search: ((Randomized Controlled Trial) OR (random)) OR (RCT) | 1366798 |
| #4 | Search: (((((((abciximab) OR (c7E3 Fab)) OR (Chimeric 7E3 Fab)) OR (Fab, Chimeric 7E3)) OR (ReoPro)) OR (Clotinab)) OR (CentoRx)) AND ((((ST Elevation Myocardial Infarction) OR (ST Segment Elevation Myocardial Infarction)) OR (ST Elevated Myocardial Infarction)) OR (STEMI)) AND (((Randomized Controlled Trial) OR (random)) OR (RCT)) | 271 |

2. EMBASE search strategy

| # | Searches | Results |
| --- | --- | --- |
| #1 | 'abciximab':ti,ab,kw OR 'c7e3 fab':ti,ab,kw OR 'chimeric 7e3 fab':ti,ab,kw OR 'fab, chimeric 7e3':ti,ab,kw OR 'reopro':ti,ab,kw OR 'clotinab':ti,ab,kw OR 'centorx':ti,ab,kw | 10525 |
| #2 | 'st elevation myocardial infarction':ti,ab,kw OR 'st segment elevation myocardial infarction':ti,ab,kw OR 'st elevated myocardial infarction':ti,ab,kw OR  'stemi':ti,ab,kw | 54652 |
| #3 | 'randomized controlled trial':ti,ab,kw OR 'random':ti,ab,kw OR 'RCT':ti,ab,kw | 1095143 |
| #4 | #1 AND #2 AND #3 | 244 |

3. Cochrane Library search strategy

| # | Searches | Results |
| --- | --- | --- |
| #1 | (abciximab):ti,ab,kw OR (c7E3 Fab):ti,ab,kw OR (Chimeric 7E3 Fab):ti,ab,kw OR (Fab, Chimeric 7E3):ti,ab,kw OR (ReoPro):ti,ab,kw OR (Clotinab):ti,ab,kw OR (CentoRx):ti,ab,kw | 964 |
| #2 | (ST Elevation Myocardial Infarction):ti,ab,kw OR (ST Segment Elevation Myocardial Infarction):ti,ab,kw OR (ST Elevated Myocardial Infarction):ti,ab,kw OR (STEMI):ti,ab,kw | 7012 |
| #3 | (randomized controlled trial):ti,ab,kw OR (random):ti,ab,kw OR (RCT):ti,ab,kw | 690148 |
| #4 | #1 AND #2 AND #3 | 143 |

4. Web of science search strategy

| # | Searches | Results |
| --- | --- | --- |
| #1 | TS=(abciximab OR c7E3 Fab OR Chimeric 7E3 Fab OR Fab, Chimeric 7E3 OR ReoPro OR Clotinab OR CentoRx) | 4067 |
| #2 | TS=(ST Elevation Myocardial Infarction OR ST Segment Elevation Myocardial Infarction OR ST Elevated Myocardial Infarction OR STEMI) | 38547 |
| #3 | TS=(randomized controlled trial OR random OR RCT) | 1306508 |
| #4 | #1 AND #2 AND #3 | 141 |

5. Articlel searched by the clinicalTrials.gov

Study title: Deferred stenting in patients with anterior wall STEMI (recruiting).

Supplementary Table 2. Summary of GRADE evidence quality for outcomes.

Abbreviations: MI: myocardial infarction
